# Two Approaches of ‘Proactive Consultation’: Towards Well-Functioning Clinical Ethics Consultation

**DOI:** 10.1007/s41649-024-00302-8

**Published:** 2024-08-15

**Authors:** Atsushi Kogetsu, Jungen Koimizu

**Affiliations:** 1 Department of Biomedical Ethics and Public Policy, Graduate School of Medicine, Osaka University, Osaka, Japan; 2 Department of Plastic and Reconstructive Surgery, Japanese Red Cross Society Kyoto Daini Hospital, Kyoto, Japan; 3 Department of Plastic and Reconstructive Surgery, Kyoto Prefectural University of Medicine, Kyoto, Japan

**Keywords:** Proactive ethics consultation, Clinical ethics consultation, Healthcare ethics consultation, Clinical ethics, Inter-professional ethics rounds, Patient note review

## Abstract

In recent years, the global need for clinical ethics consultation services (CECS) has increased to address ethical challenges, dilemmas, and moral distress in clinical environments. In Japan, many hospitals have introduced CECS over the past decade, but few such services work effectively because of the small number of consultations. To address this, we propose two proactive ethics consultation methods: inter-professional ethics rounds and patient note reviews. This paper provides a detailed explanation of these methods, complete with scenarios based on actual cases. These methods can make CECS ‘well-functioning’ by shifting the starting points of consultation from consultees to CECS providers. We then examine the impact and value of proactive ethics consultation as well as four critical factors for its success including attitude, positioning, and competency of proactive consultation teams. We believe our suggestions will provide valuable insights for future clinical ethics consultations and stimulate academic debate about what constitutes a ‘well-functioning’ CECS.

## Introduction

In recent years, the significance of ethics in healthcare has become apparent. The shift from paternalism to patient-centred care, along with an increased emphasis on shared decision-making, often leads to ethical dilemmas in decision-making (Munthe et al. 2012). Further, recent advancements in medical science have complicated matters, offering more healthcare options than ever before. One method to address these clinical ethics issues is through clinical ethics consultation. This is defined as a range of individual and group services that assist patients, families, surrogates, healthcare professionals, and other stakeholders in resolving concerns and conflicts about value issues that arise in healthcare (Tulsky and Fox 1996). It is particularly useful in specific situations, such as end-of-life healthcare decisions, especially when patients’ decision-making capacity is questionable, when there is a conflict between healthcare professionals and family members because of unknown patient wishes, or when the appropriateness of a surrogate decision-maker is uncertain (Tarzian and Force 2013).

In Japan, since the 2010s, there has been a growing trend for hospitals to form new committees or teams to address clinical ethics issues, which are encouraged by the Ministry of Health, Labour and Welfare’s guidelines on end-of-life decision-making and in the hospital functional assessment (Dowa et al. 2022; Takeshita et al. 2022; Nagao and Takimoto 2024). These groups, mainly composed of hospital staff, are set up to assist healthcare professionals with ethical dilemmas. While ethics consultation systems are becoming more common, questions about their effectiveness persist, echoing concerns raised internationally (Cederquist et al. 2021). These systems seem to work well in clear-cut situations that are easily recognised by many healthcare workers. However, straightforward cases are rare. Despite the presence of consultation systems, the frequency of actual consultations remains low. This may be because of a lack of awareness about the system among hospital staff. Some medical personnel are unsure about which issues warrant consultation, while others struggle to articulate their concerns about important but ambiguous matters (Cederquist et al. 2021). Existing clinical ethics consultation services (CECS) cannot address these issues and effective methods have not been established.

To address these issues, we have practiced ‘proactive ethics consultation’, in which the ethics consultation team actively engages with healthcare professionals through two methods in two respective hospitals. Existing literature introduced non-reactive ethics consultations without direct requests, such as initiating proactive interventions in the intensive care unit (ICU) and holding inter-professional reflection sessions to explore ethical dilemmas in heart failure management (Andereck et al. 2014; Brännström et al. 2019; Pavlish et al. 2019; Wirpsa et al. 2021). However, these may not strictly adhere to the traditional definition of ‘consultation’, which typically involves discussions or advice from experts, and may instead focus on reflective practice and the ethical training of healthcare staff. Contrastingly, we characterise proactive ethical consultation as a service initiated by the consultation team that centres on dialogues with consultees to address ethical challenges in healthcare and alleviate the moral distress experienced by healthcare professionals. This paper discusses our specific strategies of proactive ethics consultation and reflects on the potential advantages and insights gained from our practice. First, we introduce two methods of proactive ethics consultation with scenarios based on actual cases. Then, we discuss the impact and value of proactive ethics consultation as well as key considerations for its success.

## Two Approaches of Proactive Ethics Consultation

### Inter-Professional Ethics Rounds

The first approach is the ‘inter-professional ethics round’. For the past 4 years before the time of writing, a hospital’s clinical ethics consultation team has been visiting staff stations across most departments monthly, such as wards, ICUs, operating theatres, and others. More than 40 rounds have been made so far. They ask staff members if they have any concerns or ethical dilemmas. Previously, ward managers were the main point of contact for such queries, but now, with the clinical ethics link nurse system in place, these nurses gather cases from their respective departments to discuss with the consultation team. There is no need for them to pinpoint ethical issues beforehand. The goal of these rounds is to collectively articulate and ponder the nature of a problem, its ethical dimensions, and appropriate ways to address it. The topics cover both current and past cases. The consultation team might offer immediate solutions or propose a later meeting with involved parties, in which they will assist and provide guidance. Their advice is not mandatory; final decisions are made by the medical teams. However, this approach can improve the decision-making process by considering various ethical aspects.

Four years ago, when the inter-professional rounds were initiated, the common response to the inquiries was often a noncommittal ‘nothing in particular’. However, over the past year or two, the consultation team has noticed a shift, with many departments now presenting some form of report or seeking consultation. Box 1 illustrates a typical hypothetical scenario in which the consultant tried to understand the situation and identify the problem. Moreover, the case highlights communication challenges between doctors and nurses. Addressing these issues effectively is challenging within the conventional consultation system, which is typically reserved for clear-cut ethical problems or dilemmas.

#### Box 1. A Fictitious Scenario of Consultation in Inter-Professional Ethics Rounds

A woman in her 70s underwent successful surgery for oesophageal cancer at a hospital but subsequently developed post-operative pneumonia. Although the pneumonia was treated effectively with antimicrobial therapy, her respiratory function remained compromised, partly because of pre-existing lung disease. As a result, she frequently needed to be put back on a ventilator within a week of being weaned off it. The patient expressed distress over her inability to speak while on the ventilator. When the attending nurse sought guidance on the next steps in treatment from the doctor, she received no definitive response, leaving her uncertain about how to proceed.

### Patient Note Review

Another proactive method in ethics consultation involves reviewing patient notes, which was initiated 3 years ago (from the time of writing). This entails the clinical ethics consultation team examining the records of all the patients in departments with a high likelihood of ethical challenges, namely emergency departments and ICUs, weekly. To date, over 100 patient note reviews have been performed. The team evaluates whether patients and their families have been adequately informed, identifies any issues with the decision-making process, and determines whether there are complex ethical dilemmas that the medical team cannot resolve independently. When required, the consultation team reaches out to the medical staff and organises a multidisciplinary meeting, guided by the consultation team, to discuss the way forward.

In the hospital where the consultation team has only recently been established, there have been a limited number of requests for consultations from the medical care teams. However, charge nurses frequently reported cases involving ethical issues in healthcare to the nursing director, framing them as ‘difficulties’ rather than seeking formal ethics consultations. Since the initiation of patient note reviews in departments with a high risk of ethical issues, these reports of difficulties have increasingly turned into formal requests for ethics consultations directed to the consultation team, which engaged with the departments via patient note reviews. A representative fictional case is outlined in Box 2.

#### Box 2. A Fictitious Scenario Identified by the Patient Note Review

An 80-year-old man with mild dementia, undergoing regular haemodialysis for chronic kidney failure, was admitted to the ICU because of aspiration pneumonia and sepsis. Initially, his low blood pressure during dialysis sessions led to a reduction in their length and frequency. The situation deteriorated when septic shock exacerbated his low blood pressure, complicating the dialysis process, which necessitated the use of continuous haemodiafiltration and norepinephrine. His ability to continue with dialysis was uncertain, and his delirious state made it challenging to ascertain his treatment preferences. The nephrologist in charge planned to resume intermittent haemodialysis once the infection was under control and to re-evaluate his treatment approach. Meanwhile, an ethics consultation team, having examined his medical records, highlighted the need for a thorough reassessment of the end-of-life care policy, including not just haemodialysis but also artificial feeding and tracheal intubation. After contacting the physician, a clinical ethics meeting was convened to discuss the policy and acknowledge the importance of collecting information from all relevant parties.

## The Impact and Value of Proactive Ethics Consultation

### Shifting the Starting Points of Consultation from Consultees to CECS Providers

Previous research has highlighted various challenges facing ethics consultation, including a general lack of awareness about the consultation system and a low rate of its use. Cederquist et al. (2021) discovered through a survey at their institutions that the underuse of ethics consultation services often stems from frontline staff not recognising the need to seek advice for the ethical issues they encounter. Additionally, some staff members were not even aware that an ethics consultation system was in place, despite its accessibility for many years. To address these problems, it is essential to implement announcements and educational initiatives for staff (Cederquist et al. 2021).

In traditional ethics consultation systems, the process begins when the medical team requests guidance. Because the ethics consultation team remains in a passive role, waiting for these requests, the effectiveness of the service depends on the medical team’s awareness, attitude and action. This approach is known as ‘reactive ethics consultation’. Contrastingly, ‘proactive ethics consultation’, the two methods we discussed, involves the ethics team actively reaching out to the medical staff. This proactive stance not only uncovers ethical issues that the medical team may not have recognised but also raises awareness about the existence and benefits of the ethics consultation service. The distinct benefits and challenges of each method are explored further in subsequent sections.

### Advantages and Challenges of the Inter-Professional Ethics Rounds

Inter-professional ethics rounds offer considerable benefits by enabling medical care teams to articulate concerns they may be conscious of but have not yet expressed through discussions with consultation teams. These sessions are particularly useful for uncovering potential conflicts and confrontations within teams regarding medical treatment and care, which are not easily discernible from patient notes. Direct communication often brings these issues to the surface. Further, even when medical care teams do not initially perceive an ethical problem, ethical considerations are frequently relevant, and adopting ethical perspectives can prove beneficial.

Inter-professional ethics rounds serve as a valuable educational tool for healthcare providers, promoting a ‘culture’ of clinical ethics. By engaging in dialogue to clarify problems, participants gain practical insights into the ethical dilemmas present in clinical settings and learn strategies for addressing them. Regular participation in ethics rounds helps healthcare professionals to appreciate the diversity of ethical issues inherent in daily medical practice and care, highlighting the importance of recognising, sharing, and tackling these issues. The development of this ethical ‘culture’ is evidenced by the increasing number of cases brought forward for discussion during ethics rounds over time.

While patient note reviews are specific to individual patients, ethics rounds are facilitated by the responsible staff member. One advantage of ethics rounds is their ability to tackle ethical issues not directly related to specific cases; however, a drawback is their reliance on the leading staff member’s ability to recognise and sensitively handle ethical matters, which means comprehensiveness is not always assured. Although the educational advantages previously mentioned can somewhat alleviate this issue, integrating ethics rounds with other methods could enhance their effectiveness.

### Advantages and Challenges of the Patient Note Review

The patient note review stands out because it adopts a thorough approach that considers the risk of ethical dilemmas. This method identifies ethical concerns, whether considerable or minor, that may go unreported by healthcare teams for various reasons. Ethical issues that are contentious or carry a high legal risk should be tackled not only by healthcare teams but also be part of the medical institution’s policy discussions. Proactive engagement from ethics consultation teams, using this all-encompassing strategy, can avert the escalation of challenging situations into medical disputes.

Moreover, reviewing patient notes offers a chance to enhance healthcare professionals’ awareness of ethical considerations. Several ethical dilemmas that warrant review may go unreported to ethics consultation services because medical staff fail to recognise the necessity for such consultations (Cederquist et al. 2021). Standard ethics rounds and reactive consultations are ineffective for issues that medical staff do not acknowledge. Thus, by bringing these issues to the attention of consultation teams via patient note reviews, an educational effect on medical staff can be achieved. For instance, in the example in Box 2, after finding the ethics consultation service helpful, the attending physician began to proactively seek the team’s advice on similar cases. This approach may encourage more staff to appreciate and actively engage with consultation services.

However, this comprehensive approach presents certain challenges. Although confined to high-risk departments, the detailed review of patient notes is labour-intensive. Streamlining the assessment process to focus on higher-risk cases could be beneficial (Pavlish et al. 2019).

## Four Key Considerations for Successful Proactive Ethics Consultation

### Ensure Psychological Safety

When initiating proactive consultations, it is crucial to foster an environment in which individuals feel at ease to speak freely. For instance, during ethics rounds, some staff preface with ‘I do not think this is about ethics…’. In response, reassuring them with ‘You do not have to focus on ethical issues. Feel free to discuss whatever is on your mind’ can help them relax and open up. Active listening and empathy are vital in these conversations, and it is important to always show respect for the healthcare professionals directly involved in patient treatment and care. These skills are recognised as core competencies for ethics consultants (Tarzian et al. 2013; Wasson et al. 2019). However, in proactive consultation, the consultant’s approach is even more meaningful, because the dialogue begins without a request from the medical care teams. This approach is key to ensuring the consulters’ psychological safety and fostering a trusting relationship between the medical care teams and the consultation teams.

### Do not Pursue the ‘Answer’ Too Much

In reactive consultations, medical teams have identified specific needs by the time they request assistance, aiming to address these needs, often seeking a solution to an issue or validation for their decision-making process (McClimans et al. 2019). Contrastingly, proactive consultations emerge without an immediate need and may not require a direct response. For instance, during ethics rounds, not every consultation with medical teams demands a ‘prescription’. At times, healthcare professionals may find it sufficient to engage in conversation with consultants, or they may benefit from the structured thinking that arises from the dialogue. This means that even when proactive consultations identify problems, a solution is not the sole expectation from those seeking advice.

### Be Conscious of Where the Teams Stand

It is undeniable that medical teams might feel their work is under scrutiny, particularly when patient notes are reviewed. To avoid any negative reactions, it is crucial to handle staff emotions with care and adopt a supportive stance when discussing ethical issues identified by consultation teams. Moreover, allowing consultants, who are not part of the direct medical care team, to access patient notes without the medical team’s consent can raise concerns about patient privacy. The role and duties of the consultation team within the hospital should be clearly explained and justified beforehand.

### Secure Resources and Cultivate Skills

Limitations of our methods include securing resources and consultation providers’ competency. Proactive consultations require more time and effort than reactive ones. Additionally, during ethics rounds, consultation teams lack the opportunity to prepare discussions beforehand, necessitating the ability to offer spontaneous advice. This includes not only the capacity to provide specific, ethically informed advice and practical recommendations but also the facilitation skills required to help consulters identify issues and appropriate responses during the dialogue. Establishing a system that prevents proactive consultation from becoming reliant on the expertise of a single skilled consultant, akin to ‘craftsmanship’, is crucial. Developing the consultation team’s skills is vital, with on-the-job training and well-designed role-play being effective methods to achieve this (Udagawa and Takimoto 2022).

## Conclusion

In this paper, we proposed proactive ethics consultation as a key component of an effective CECS. We introduced two specific strategies—inter-professional ethics rounds and patient note review—illustrating them with detailed methods and hypothetical cases. We explored their potential effects and value and outlined essential considerations for their practical application. Our approach to proactive ethics consultation is distinct from ‘proactive ethics intervention’ and sessions focused on healthcare professionals’ reflection and ethics education, which have been previously described as proactive. The main goal of our consultation is to address ethical dilemmas in healthcare settings and alleviate moral distress among healthcare workers. The concept of moral distress has traditionally been defined as a dilemma between moral judgement and reality. In addition, Morley et al. reported that internal constraints such as lack of assertiveness and the ethical climate of the workplace can also cause moral distress. Proactive ethics consultation can reduce moral distress by providing consultation to resolve dilemmas and avoid the causes of internal constraints (Morley et al. 2019). While it is essential to maintain the balance between securing resources and assuring comprehensiveness, our methods can make CECS ‘well-functioning’ by proactively approaching potential ethical issues and cultivating a ‘culture’ of clinical ethics. We believe this paper offers valuable practical guidance for those involved in CECS and contributes to the scholarly discussion on the topic. We expect this proactive consultation approach to be adopted in various settings and its effectiveness confirmed by future empirical studies in these areas.
